# Cytotoxicity of norstictic acid derivatives, a depsidone from *Ramalina anceps* Nyl.

**DOI:** 10.55730/1300-0527.3694

**Published:** 2024-08-05

**Authors:** Danielle BOGO, Isabel Máximo C. ALCÂNTARA, Glaucia B. ALCANTARA, Ana Camila MICHELETTI, Neli K. HONDA, Maria de Fátima C. MATOS

**Affiliations:** 1 Laboratory of Molecular Biology and Cell Culture, Federal University of Mato Grosso do Sul, Campo Grande, MS, Brazil; 2 Institute of Chemistry, Federal University of Mato Grosso do Sul, Campo Grande, MS, Brazil

**Keywords:** Cytotoxic activity, selectivity index, alkyl derivatives, lichens, phenols

## Abstract

Structural modifications in lichen phenolic compounds have been one of the tools to potentiate their biological activity. In the present work, seven alkyl derivatives of norstictic acid were prepared and evaluated against eight cell lines. Norstictic acid was isolated from the lichen *Ramalina anceps* and the alkyl derivatives were obtained through reactions with alcohols. Cytotoxicity was evaluated against the 786-0 (kidney carcinoma), MCF7 (breast carcinoma), HT-29 (colon carcinoma), PC-03 (prostate carcinoma), HEP2 (laryngeal carcinoma), B16-F10 (murine melanoma), UACC-62 (human melanoma), and NIH/3T3 (mouse embryonic fibroblast) cell lines using the sulforhodamine B assay. Norstictic acid exhibited poor activity, while the 8′-*O*-*n*-butyl-norstictic acid and 8′-*O*-*sec*-butyl-norstictic acid derivatives showed potential activity (GI_50_ values of 6.37–45.0 μM and 6.8–52.40 μM, respectively) and high selectivity (selectivity index (SI) values of 13.88–98.11 and SI 11.30–87.40, respectively) against all tumor cells. The 8′-*O*-*n*-hexyl-norstictic acid showed good activity (5.96–9.53 μM) and moderate selectivity (SI 9.2–5.76) against MCF7, HT-29, PC-03, and HEP2 cells, while 8′-*O*-isopropyl-norstictic acid demonstrated high activity and selectivity against PC-03 cells (GI_50_ 1.28 μM and SI 33.8), and was highly active but moderately selective against UACC, HEP2, and B16-F10 cells (GI_50_ 6.2, 7.78, and 9.65 μM; SI 7.0, 5.5, and 4.5, respectively). Additionally, 8′-*O*-*n*-pentyl- and 8′-O-*tert*-butyl-norstictic acids were active and selective against PC-03 cells (GI_50_ 8.77 and 7.60 μM; SI 6.53 and 5.0, respectively). Chemometric analysis revealed a clear relationship between all compounds and their biological activities. The insertion of a four-carbon alkyl chain (*n*-butyl and *sec*-butyl) produced potentially active compounds on all tested tumor cells.

## 1. Introduction

The substances of the depside and depsidone classes represent most of the compounds resulting from lichen metabolism. Depsides are esters derived from orsellinic or *β*-methyl orsellinic acids, or both, while depsidones result not only from esterification between two aromatic units (orsellinic acid or *β*-methyl orsellinic acid) but also from the etherification of these units. The structural diversity of depsides and depsidones is due to the occurrence of oxidative processes in the methyl located at the positions 3 and 3′ of the aromatic ring in CH_2_OH, CHO or COOH; methylation of hydroxyls at the positions 2, 4 or 2′ (OCH_3_); and carboxyl esterification at the position 1′ (turning into methyl ester or orsellinic ester, which results in the formation of tridepsides) [1,2].

Many of the lichen-produced phenolic compounds are potentially active against microorganisms, serving as inhibitors of various enzymes and tumor cell growth, among other functions [3–6]. The antitumor activity of lichen extracts and compounds isolated from lichens has been extensively evaluated [7–9]. Among lichen depsidones, norstictic acid has been assayed for its antitumor activity. Brandão et al. [10] evaluated the effects of norstictic acid against UACC-62 human melanoma cells and determined the growth inhibition effect (GI_50_ 32.9 μg/mL, 88.0 μM), with stronger selectivity (SI > 7.6) than doxorubicin (SI 1.2). According to Ebrahim et al. [11], norstictic acid significantly suppressed the proliferation, migration, and invasion of TNBC MDA-MB-231 cells, while demonstrating minimal toxicity to nontumorogenic MCF-10A mammary epithelial cells. The in vivo effect of norstictic acid treatment on the growth of MDA-MB-231/GFP cells implanted into athymic female nude mice suggested the tolerability and significant inhibitory effect. We evaluated the cytotoxic effect of the norstictic acid on 786-0, MCF7, HT-29, PC-3, HEP2, and NIH/3T3 cells and the GI_50_ varied from 156.9 ± 7.46 to 915.6 ± 91.27 μM and it was selective only for HEP2, MCF7, and PC-3 cells (SI 4.4, 4.3, and 3.6, respectively) [12].

Structural modification of phenolic compounds, including some obtained from lichens, has demonstrated promising results in potentiating biological activity. Gallic acid and alkyl gallates have shown several activities, such as antitumoral, antimicrobial, antioxidant, and others. Despite exhibiting antitumor activity, the alkyl gallates with chains comprising less than eight carbon atoms are less active than gallates with 8–14 carbons atoms [13]. Bazin et al. [14] prepared nine derivatives of usnic acid by amine conjugation, aiming to improve the activity of usnic acid. The polyamine derivatives showed a significant cytotoxicity against L1210 cells. Kumar and Müller [15] synthesized a series of 48 barbatic and diffractaic acids analogues and evaluated them as inhibitors of leukotriene B_4_ (LTB_4_) biosynthesis and antiproliferative agents. The compound ethyl 4-*O*-demethylbarbatate was the most active compound. Alkyl orsellinates, prepared by alcoholysis of lecanoric acid, were examined for various biological activities, proving to be a very promising class of compounds. They showed good activity on brine shrimp lethality assay [16], antioxidant activity [17], were active against Hep2, MCF7, 786-0, and B16-F10 cell lines [18], and can act as tyrosinase modulators, with inhibition dependent on chain elongation [19]. The effects of alkyl orsellinates, along with alkyl 2-hydroxy-4-methoxy-6-*n*-pentylbenzoates, were also evaluated on the germination and growth of *L. sativa* and *A. cepa*. The results indicated that several compounds could serve as models for the development of new herbicides [20,21]. Micheletti et al. [22] prepared hybrid lichexantone-THC (benzopyran group) derivatives, which were highly effective against Gram-positive bacterial strains. Considering the low cytotoxic activity of norstictic acid against a panel of tumor cells [12] and using structural modification as a strategy to produce potentially active compounds, we carried out the alkylation reactions on norstictic acid. We report here the cytotoxic activity of seven 8′-*O*-alkyl-norstictic acid derivatives against HT-29 (colon carcinoma), 786-0 (kidney carcinoma), MCF7 (breast carcinoma), HEP_2_ (laryngeal carcinoma), PC-03 (prostate carcinoma), B16-F10 (murine melanoma), UACC-62 (human melanoma), and NIH/3T3 (mouse embryonic fibroblast) cell lines.

## 2. Material and methods

### 2.1. General procedures

The solvents used were PA grade (Merck and Synth), previously distilled and dried with Na_2_SO_4_, when necessary. Thin-layer chromatography (TLC) was performed on precoated silica gel 60 GF_254_ plates (Merck). Spots were visualized by spraying the plates with a 10% H_2_SO_4_-methanol solution, followed by heating. Si-gel (Merck, 230–400 mesh) was used in chromatography columns. NMR spectroscopy was performed using a Bruker DPX-300 spectrometer, with the solvent serving as an internal reference. Mass spectra were obtained with a Shimadzu QP 5050 spectrometer using direct injection and electron impact at 70 eV. The melting points were determined using a Uniscience Melting Point apparatus without corrections.

### 2.2. Lichens

*Ramalina anceps* Nyl., was obtained from home decor stores. The identification was conducted by Prof. Dr. Marcelo P. Marcelli (Institute of Environmental Research, São Paulo State Government). The voucher specimen is deposited at the Campo Grande Herbarium, UFMS (CGMS 49839). This species is registered on the SisGen platform (entry A4CE261).

### 2.3. Extraction and isolation of norstictic acid

The lichen thalli (50.0 g) was powdered and extracted first with dichloromethane exhaustively, at room temperature, and then with acetone under the same conditions. The extracts were concentrated under vacuum using a rotary evaporator, and the residues obtained were chromatographed on TLC with the eluent toluene:dioxane:acetic acid (180:45:5 v/v/v). The concentrated acetonic extract containing norstictic acid was treated with small portions of acetone in an ice bath, followed by centrifugation at 3000 rpm for 5 min. The colored supernatant was removed, and the process was repeated until the compound was obtained in a pure form. The structure was confirmed through NMR analyses. Norstictic acid: white solid [yield 2.1 g (4.2%) mp. 285–287 °C (dec.)]. ^1^H-NMR (DMSO-d_6_) *δ*: 2.20 (3H, s, CH_3_-9′), 2.45 (3H, s, CH_3_-8), 6.78 (1H, d, *J* = 7.5, H-8′), 6.87(1H, s, H-5), 8.30 (1H, d, *J* = 7.5, OH-8′), 10.26 (1H, s, OH-2′), 10.45 (1H, s, CHO), 12.06 (1H, s, OH-4). ^13^C-NMR (DMSO-*d*6) *δ*: 9.7 (C-9′), 21.5 (C-8), 95.0 (C-8′), 109.2 (C-1′), 110.7 (C-3), 111.9 (C-1), 117.4 (C-5), 121.0 (C-3′), 135.9(C-6′), 137.4 (C-5′), 147.9 (C-4′), 152.0 (C-2′), 152.4 (C-6), 160.4 (C-7), 163.6 (C-7′), 164.0 (C-4), 166.2 (C-2), 192.7 (C-9) [1].

### 2.4. Structural modifications

**Figure 1:**
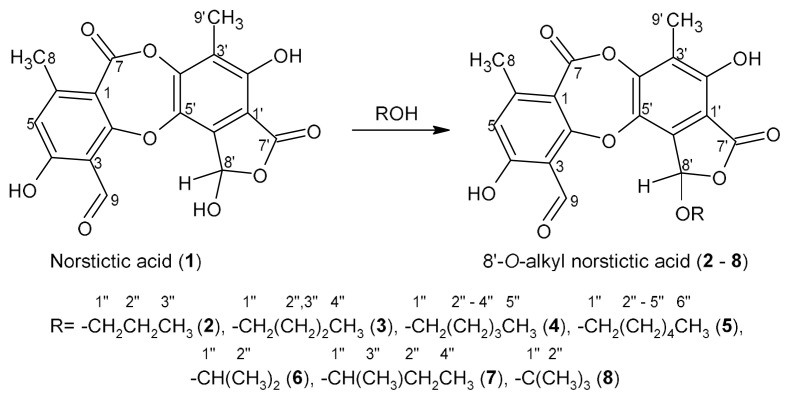
Representation of the reactions of norstictic acid with alcohols.

The preparation of 8′-*O*-alkyl derivatives of norstictic acid was carried out by treating norstictic acid (200 mg) with excess of alcohols, including *n*-propanol, *iso*-propanol, *n*-butanol, *sec*-butanol, *tert*-butanol, *n*-pentanol, and *n*-hexanol (50 mL), under heating at the boiling temperature of each alcohol (Figure 1), according to the methodology described by Micheletti et al. [23]. Reactions were monitored by TLC. The product of each reaction was purified using column chromatography with 230–400 mesh silica gel and hexane-acetone gradient as the eluent. To confirm the structure of the 8′-*O*-alkyl-norstictic acid derivatives, ^1^H, ^13^C, and DEPT 135 NMR and mass spectra were obtained. The chemical shifts of atoms from the alkyl chains were compared with those described by Lopes et al. [16] and Micheletti et al. [23].

8′-*O*-*n*-propyl-norstictic acid (**2**). White solid, mp. 183 °C. ^1^H-NMR (CDCl_3_) δ: 2.30 (3H, s, ArCH_3_-9′), 2.51 (3H, s, ArCH_3_-8), 6.49 (1H, s, H-8′), 6.72 (1H, s, ArH-5), 10.44 (1H, s, ArCHO), [R = 0.88 (3H, t, *J* = 7.5 Hz, -CH_3_-3″), 1.60 (2H, m, -CH_2_-2″), 3.75 (2H, t, *J* = 7.5 Hz, -CH_2_-1″)]. ^13^C-NMR (CDCl_3_) δ: 9.2 (C-9′), 22.4 (C-8), 101.1 (C-8′), 107.9 (C-1′), 110.3 (C-3), 111.9 (C-1), 118.1 (C-5), 121.7 (C-3′), 131.5 (C-6′), 138.5 (C-5′), 149.4 (C-4′), 152.8 (C-2), 153.9 (C-6), 160.1 (C-7), 164.1 (C-4), 165.6 (C-2), 169.2 (C-7′), 192.0 (C-9), [R = 10.2 (C-3″), 22.5 (C-2″), 71.7 (C-1″)]. EI-MS m/z: 414. Yield: 60%.

8′-*O*-*n*-butyl-norstictic acid (**3**). White solid, mp. 169 °C. ^1^H-NMR (CDCl_3_) δ: 2.30 (3H, s, ArCH_3_-9′), 2.52 (3H, s, ArCH_3_), 6.49 (1H, s, H-8′), 6.73 (1H, s, ArH-5), 10.45 (1H, s, ArCHO), 12.09 (1H, s, ArOH-2′, [R = 0.90 (3H, t, *J* = 6.0 Hz, -CH_3_-4″), 1.30 (2H, m, -CH_2_-3″), 1.55 (2H, m, -CH_2_-2″), 3.78 (2H, t, *J* = 6.0 Hz, -CH_2_-1″)]. ^13^C-NMR (CDCl_3_) δ: 9.5 (C-9′), 22.3 (C-8), 101.4 (C-8′), 108.6 (C-1′), 110.3 (C-3), 111.9 (C-1), 118.1 C-5), 121.7 (C-3′), 131.5 (C-6′), 138.5 (C-5′), 149.4 (C-4′), 152.8 (C-2), 153.9 (C-6), ), 160.1 (C-7), 164.0 (C-4), 165.6 (C-2), 169.2 (C-7′), 192.9 (C-9), [R = 13.9 (C-4″), 19.2 (C-3″), 31.4 (C-2″), 70.2 (C-1″)]. EI-MS m/z: 428. Yield: 46.8%.

8′-*O*-*n*-pentyl-norstictic acid (**4**). White solid, mp. 175 °C. ^1^H-NMR (CDCl_3_) δ: 2.30 (3H, s, ArCH_3_-9′), 2.52 (3H, s, ArCH_3_-8), 6.49 (1H, s, H-8′), 6.72 (1H, s, ArH-5), 10.45 (1H, s, ArCHO), 12.08 (1H, s, ArOH-2′), [R = 0.89 (3H, t, *J* = 6.0 Hz, -CH_3_-5″), 1.35 (4H, m, CH_2_-4″, -CH_2_-3″), 1.59 (2H, m, -CH_2_-2″) 3.77 (2H, t, *J* = 6.0 Hz, -CH_2_-1″)]. ^13^C-NMR (CDCl_3_) δ: 9.2 (C-9′), 22.3 (C-8), 101.1 (C-8′), 108.0 (C-1′), 110.3 (C-3), 111.9 (C-1), 118.1 (C-5), 121.7 (C-3′), 131.6 (C-6′), 138.4 (C-5′), 149.4 (C-4′), 152.8 (C-2′), 153.9 (C-6), 160.0 (C-7), 164.0 (C-4), 165.6 (C-2), 169.2 (C-7′), 192.9 (C-9), [R = 13.9 (C5″), 22.3 (C-4″), 27.8 (C-3″), 28.8 (C-2″), 70.3 (C-1″)]. EI-MS m/z: 442. Yield: 54%.

8′-*O*-*n*-hexyl-norstictic acid (**5**). White solid, mp. 178 °C. ^1^H-NMR (CDCl_3_) δ: 2.30 (3H, s, ArCH_3_-9′), 2.44 (3H, s, ArCH_3_-8), 6.49 (1H, s, H-8′), 6.72 (1H, s, ArH-5), 10.44 (1H, s, ArCHO), 12.10 (1H, s, ArOH-2′), [R = 0.85 (3H, t, *J* = 6.0 Hz, -CH_3_-6″), 1.25 (6H, s, -CH_2_-5″, -CH_2_-4″, -CH_2_-3″), 1.55 (2H, m, -CH_2_-2″), 3.77 (2H, t, *J =* 6.0 Hz, -CH_2_-1″)]. ^13^C-NMR (CDCl_3_) δ: 9.2 (C-9′), 22.3 (C-8), 101.1 (C-8′), 107.9 (C-1′), 110.3 (C-3), 111.9 (C-1), 118.10 (C-5), 121.7 (C-3′), 131.5 (C-6′), 138.5 (C-5′), 149.4 (C-4′), 152.8 (C-2′), 153.9 (C-6), 160.1 (C-7), 164.2 (C-4), 169.2 (C-7′), 192.9 (C-9). [R = 13.9 (C6″), 22.4 (C-5″), 25.4 (C-4″), 29.1 (C-3″), 31.3 (C-2″), 70.3 (C-1″)]. EI-MS m/z: 456. Yield: 52%.

8′-*O*-isopropyl-norstictic acid (**6**). White solid, mp. 185 °C. ^1^H-NMR (CDCl_3_) δ: 2.30 (3H, s, ArCH_3_-8), 2.52 (3H, s, ArCH_3_-9′),6.57 (1H, s, H-8′), 6.72 (1H, s, ArH-5), 10.51 (1H, s, ArCHO), [R = 1.25 (3H, d, 6 Hz, -CH_3_-2″), 1.40 (3H, d, 6 Hz, -CH_3_-2″), 4.25 (1H, m *J* = 6.0 Hz, -CH-1″)]. ^13^C-NMR (CDCl_3_) δ: 9.2 (C-9′), 20.8 (C-8), 99.3 (C-8′), 108.0 (C-1′), 110.3 (C-3), 111.9 (C-1), 118.1 (C-5), 121.6 (C-3′), 131.9 (C-6′), 138.5 (C-5′), 149.3 (C-4′), 154.0 (C-2′), 154.1 (C-6), 160.1 (C-7), 164.2 (C-4), 165.6 (C-2), 169.3 (C-7′), 193.1 (C-9). [R = 22.4, 22.8 (2× C-2″), 73.0 (C-1″)]. EI-MS m/z: 414. Yield: 44.3%.

8′-*O*-*sec*-butyl-norstictic acid (**7**). White solid, mp. 170 °C. ^1^H-NMR (CDCl_3_) δ: 2.30 (3H, s, ArCH_3_-9′), 2.52 (3H, s, ArCH_3_-8), 6.58 (1H, s, ArH-5), 6.72 (1H, s, H-8′), 10.05 (1H, s, ArCHO), 12.11 (1H, s, ArOH-2′), [R = 0.85 (3H, t, *J* = 7.4 Hz, -CH_3_-4″), 1.58 (3H, d, 6.48 Hz, -CH_3_-3″), 1.70 (2H, m, -CH_2_-2″), 3.98 (1H, m, -CH-1″)]. ^13^C-NMR (CDCl_3_) δ: 9.2 (C-9′), 20.0 (C-8), 99.0 (C-8′), 108.0 (C-1′), 110.3 (C-3), 110.4 (C-1), 118.0 (C-5), 121.5 (C-3′), 131.9 (C-6′), 138.6 (C-5′), 121.6 (C-3′), 131.9 (C-6′), 138.6 (C-5′), 149.3 (C-4′), 152.8 (C-2′), 154.0 (C-6), 160.1 (C-7), 164.2 (C-4), 165.5 (C-2), 169.3 (C-7′), 192.9 (C-9), [R = 9.6 (C-4″), 17.8 (C-3″), 29.5 (C-2″), 78.6 (C-1″)]. EI-MS m/z: 428. Yield: 45.2%.

8′-*O*-*tert*-butyl-norstictic acid (**8**). White solid, mp. 196 °C. ^1^H-NMR (CDCl_3_) d: 2.35 (3H, s, ArCH_3_-9′), 2.50 (3H, s, ArCH_3_-8), 6.60 (1H, s, ArH-5), 6.70 (1H, s, H-8′), 10.05 (1H, s, ArCHO), 12.10 (1H, s, ArOH-2′), [R = 1.60 (9H, s, CH_3_-2″)]. ^13^C-NMR (CDCl_3_) δ: 9.3 (C-9′), 22.5 (C-8), 101.0 (C-8′), 108.1 (C-1′), 110.3 (C-3), 110.4 (C-1), 118.0 (C-5), 122.0 (C-3′), 134.5 (C-6′), 137.8 (C-5′), 149.3 (C-4′), 152.6 (C-2′), 154.4 (C-6), 160.1 (C-7), 164.4 (C-4), 165.3 (C-2), 169.2 (C-7′), 192.5 (C-9), [R = 27.8 (C-2″), 76.5 (C-1″)]. EI-MS m/z: 428. Yield: 48%.

### 2.5. *In vitro* cytotoxic activity

MCF7 (ATCC HTB-22, breast adenocarcinoma), 786-0 (ATCC CRL-1932, renal cell adenocarcinoma), PC-03 (ATCC CRL-1435, prostatic adenocarcinoma), HT-29 (ATCCHTB-38, colorectal adenocarcinoma), and HEP2 (ATCC CCL-23, laryngeal carcinoma) cells donated by Dr. João Ernesto de Carvalho (CPQBA – UNICAMP), and NIH/3T3 cells (ATCC CRL-1658, mouse embryonic fibroblast), purchased from the Rio de Janeiro Cell Bank, were used for the evaluation of cytotoxic activity. Cell maintenance and treatment were performed as described by Freshney [24]. The sulforhodamine B (SRB) assay was carried out according to Brandão et al. [10] and Bogo et al. [12].

### 2.6. Selectivity index

The selectivity index (SI) measures a compound’s ability to target a neoplastic cell line rather than a normal cell line, indicating its potential for use in clinical trials. In the present study, the SI of each substance was calculated as the quotient of its GI_50_ value for normal NIH/3T3 cells divided by the GI_50_ value for a neoplastic cell line. SI values greater than 3.0 were considered significant [25].

### 2.7. Chemometric treatment

Principal component analysis (PCA) was applied on the biological activity data for dimensionality reduction of the results. Two chemometric matrices were evaluated. First, the GI_50_ values (the concentration required to inhibit the growth of cancerous cell lines by 50%) were analyzed to outline the general behavior of each compound against seven cancerous cell lines: HT-29, 786-0, MCF7, HEP_2_, PC-03, B16-F10, and UACC-62. Finally, the selectivity index (SI) for the compounds on all cell lines was used to understand the selectivity of the cytotoxicity. PCA was performed using mean-centered preprocessing.

## 3. Results and discussion

Norstitic acid (**1**) was treated with linear and branched chain alcohols containing three to six carbons under reflux, yielding seven derivatives. The structure of these derivatives was elucidated by NMR spectroscopy, which confirmed the insertion of the alcohol chain at the 8′ position of (**1**) for all derivatives. The profiles of the ^1^H and ^13^C NMR spectra for all derivatives are quite similar, allowing for the observation of signals corresponding to the skeleton of the starting material and the inserted alkyl chain. The position where the reaction occurred was confirmed by the chemical shift values of the carbons and hydrogens 1″, originally linked to the hydroxyl of the alcohol, as well as the 8′ carbons. In the ^1^H NMR spectra of the derivatives, it is possible to observe signals in the range of 3.7 ppm (for linear chain derivatives), which are compatible with 1″ hydrogens. Additionally, in the ^13^C NMR spectra, signals in the range of 70 ppm are observed, which are assigned to carbons 1″. There are also signals in the range of 101 ppm, which can be attributed to the 8′ carbons of the derivatives. The deprotection of approximately 6 ppm in relation to norstictic acid (**1**) is consistent with the insertion of a substituent in this position. The chemical shift values align with those observed by Micheletti et al. [23] for the same type of derivative. Furthermore, if the transesterification reaction had occurred, with the alcohol attacking the C-7 carbonyl, the signals for group 1″ (CH_2_, CH or C, depending on the reagent) would be observed in the regions of approximately 4.3 to 5.2 ppm in the ^1^H NMR spectra and 65 to 67 ppm in the ^13^C NMR spectra for linear chains (for branched chains, a significant variation in the chemical shift values of these carbons would be expected, depending on the substituents attached to them) [16].

Norstictic acid (**1**) and its alkyl derivatives (**2)**–(**8**) were evaluated for their antiproliferative activities against several tumor cell lines. Table shows growth inhibition (GI_50_) and selectivity index (SI) values for all compounds and doxorubicin (positive control) against the tested cells. The norstictic acid (**1**) was selective against the MCF7, HEP2, PC-03, B16-F10, and UACC-62 cells (SI 4.3, 4.4, 3.6, > 4.1 and > 7.6), with the GI_50_ ranging from 88.4 to 191.2 μM. Based on the results presented in Table, we can select the compounds 8′-*O*-*n*-butyl-norstictic acid (**3**) and 8′-*O*-*sec*-butyl-norstictic acid (**7**) as potential cytotoxic agents [(6.27 ≤ GI_50_ ≤ 52.40 μM), with high selectivity indices ranging from 13.88 to 98.11 for compound (**3**) and 11.3 to 87.4 for compound (**7**)]. The 8′-*O*-*n*-pentyl-norstictic acid (**4**) demonstrated higher activity against MCF7 and PC-03 cells, with GI_50_ values of 15.53 and 8.77 μM and SI values of 3.69 and 6.53, respectively. In contrast, the 8′-*O*-*n-*hexyl-norstictic acid (**5**) derivative showed activity against MCF7, HT-29, PC-03, and HEP2 cells. Although 8′-*O*-*n*-propyl derivative (**2**) did not show activity against the tested tumor cells, the isopropyl derivative (**6**) was active against PC-03, HEP_2_, B16-F10, and UACC-62 cells (GI_50_ ranging from 1.28 to 9.65 μM and SI ranging from 4.48 to 33.8). The 8′-*O*-*tert*-butyl derivative (**8**) was active only against PC-03 cells (GI_50_ 7.60 μM and SI 5.0). All 8′-*O*-alkyl-norstictic acid derivatives were active against PC-03 cells, with compounds containing *n*-butyl and *n*-propyl moieties exhibiting GI_50_ values of 6.37 μM (SI 98.11) and 1.28 μM (SI 33.8), respectively. Although all the derivatives (**2**) to (**8**) were less active than doxorubicin against the tested cell lines, many of these compounds exhibited much higher selectivity than the drug. For example, compounds (**3**) and (**7**) are the most promising antitumor agents, demonstrating potential activity against the tested tumor cells while showing the lowest effect on 3T3 cells.

The literature reports the synthesis and biological evaluation of substances related to the derivatives described here. Micheletti et al. [23] prepared a series of compounds from salazinic acid, a depsidone isolated from the lichen *Parmotrema lichexanthonicum*, which were also tested against tumor cell lines. In this case, compounds featured two alkyl chains, and n-butyl, n-pentyl, and n-hexyl derivatives were tested on MDA/MB-435 (breast adenocarcinoma), HCT-8 (colorectal adenocarcinoma), and SF-295 (glioblastoma) cell lines. The authors obtained significant results, with IC_50_ values ranging from 1.73 to 21.66 μg/mL (3.1 to 40.93 μM). Bogo et al. [18] presented the results of cytotoxic activity of alkyl orsellinates, prepared from the depside lecanoric acid. These derivatives were evaluated against HEP_2_, MCF7, 786-0, B16-F10, and Vero cell lines, with IC_50_ ranging from 7.2 to 64.8 μg/mL (32.14 to 294.5 μM). In both cases, as observed in our study, the derivatives were more active than the starting material, showing the effectiveness of structural modification as a strategy in drug design.

**Figure 2:**
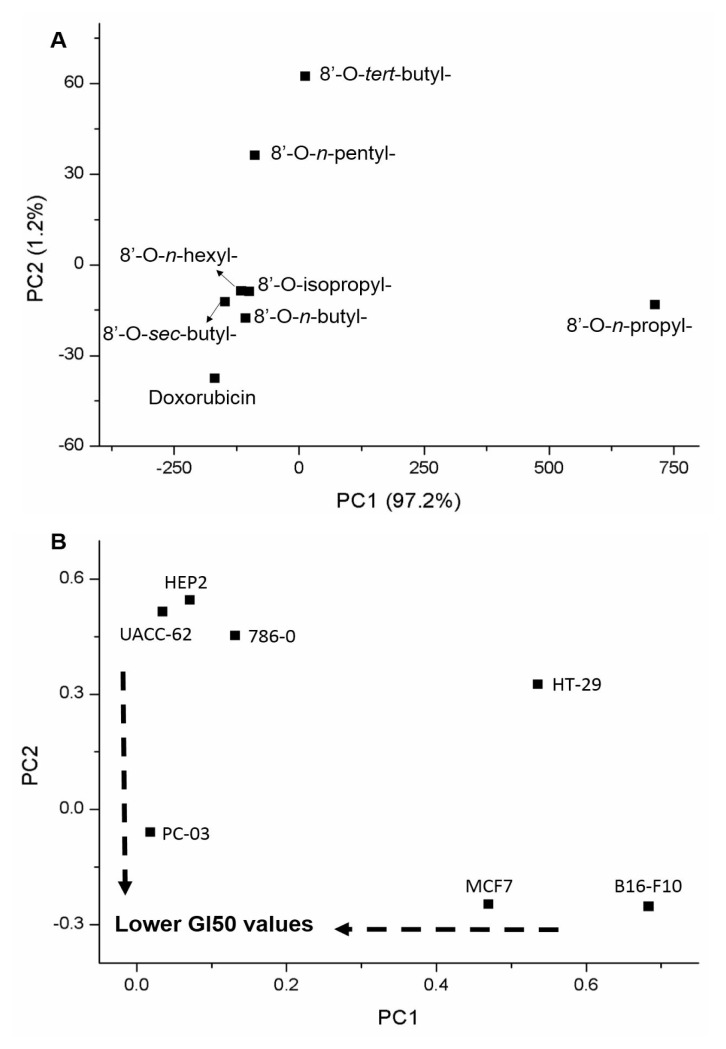
PCA score (A) and loading (B) plots from growth inhibition values (GI_50_) for all norstictic acid derivatives (8′-*O*-*n*-propyl-, 8′-*O*-*n*-butyl-, 8′-*O*-*n*-pentyl-, 8′-*O*-*n*-hexyl-, 8′-*O*-isopropyl-, 8′-*O*-*sec*-butyl-, and 8′-*O*-*tert*-butyl-norstictic acids) against the tested cancerous cell lines (HT-29, 786-0, MCF7, HEP_2_, PC-03, B16-F10, and UACC-62).

Chemometric analysis revealed a clear relationship between all compounds and their biological activities. Considering the molar concentration required to inhibit the growth (GI_50_) of cancerous cell lines by 50%, the PCA for the norstictic acid derivatives showed that 8′-*O*-*n*-butyl-, 8′-*O*-*sec*-butyl-, 8′-*O*-*n*-hexyl-, and 8′-*O*-isopropyl-norstictic acids [(**3**), (**7**), (**5**), (**6**)] were close to zero and grouped together with the doxorubicin standard. The 8′-*O*-*n*-pentyl- and 8′-*O*-*tert*-butyl-norstictic acids (**4**, **8**) were grouped on the negative PC1 axis, while the 8′-*O*-*n*-propyl-norstictic acid (**2**) was allocated to the positive PC1 axis (Figure 2A). This arrangement of the compounds on the score plot resulted from the similarities and differences in their biological activities, as indicated by the GI_50_ values (Figure 2B). The 8′-*O*-*n*-propyl-norstictic acid (**2**) exhibited a greater distance from the doxorubicin standard in the PC1, which described more than 97% of the explained variance, due to its comparatively low activity against the cancerous cell lines. The derivatives 8′-*O*-*n-*pentyl- and 8′-*O*-*tert*-butyl-norstictic acids (**4**, **8**) also exhibited low biological activity, although they were close to the doxorubicin in PC1 and distant in PC2 (Figure 2A).

**Figure 3:**
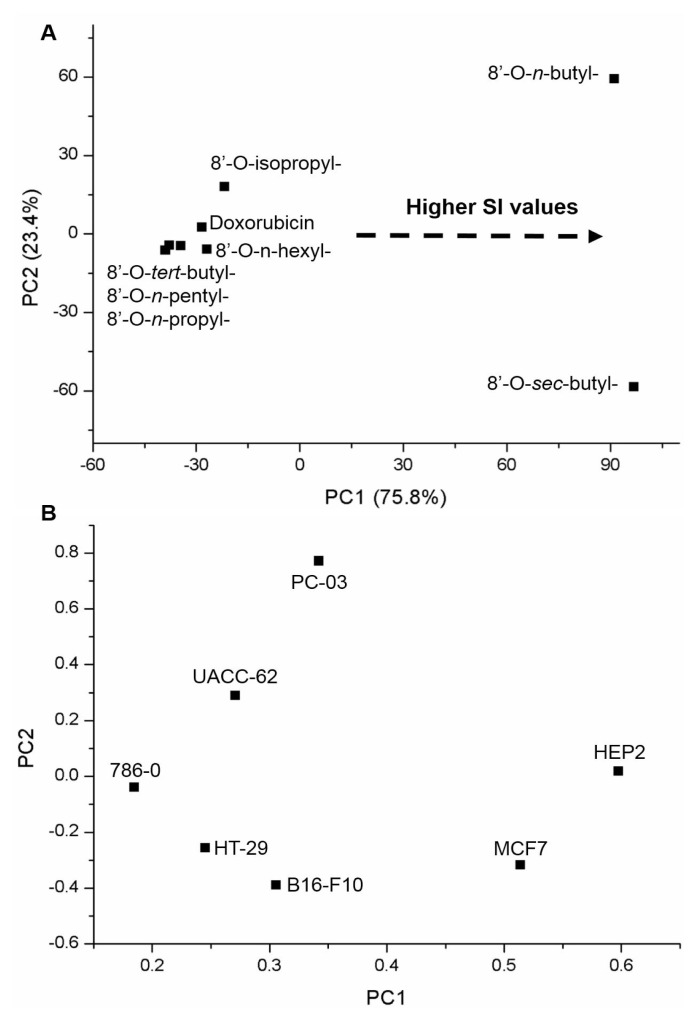
PCA score (A) and loading (B) plots from selectivity index values (SI) for all norstictic acid derivatives (8′-O-*n*-propyl-, 8′-O-*n*-butyl-, 8′-O-*n*-pentyl-, 8′-O-*n*-hexyl-, 8′-O-isopropyl-, 8′-O-*sec*-butyl-, and 8′-O-*tert*-butyl-norstictic acids) for cancerous cell lines (HT-29, 786-0, MCF7, HEP_2_, PC-03, B16-F10, and UACC-62) compared to healthy cells (3T3/NIH cell line).

In addition, the PCA of selectivity index from norstictic acid derivatives highlighted the 8′-*O*-*n*-butyl- and 8′-*O*-*sec*-butyl-norstictic acids (**3**) and (**7**) as the most different compounds on the positive PC1 axis (Figure 3A). Both compounds had the most promising biological activities due to their high specificity against cancerous cell lines when compared to healthy cells (3T3/NIH cell line). These SI values were still higher than those found for doxorubicin. The higher SI values for 8′-*O*-*sec*-butyl-norstictic acid (**7**) were observed in the MCF7, HEP2, B16-F10, and HT-29 cell lines, which allocated on the most negative PC2 axis. In contrast, the higher SI values for 8′-*O*-*n*-butyl-norstictic acid (**3**) were detected in the PC-03, HEP2, and MCF7 cell lines (Figure 3B).

These results indicated that the structural modifications to norstictic acid (**1**) structure, through the insertion of an alkyl chain at C-8′, produced compounds with potential antitumor activity, many of them were highly selective for the tested cells. Among the evaluated compounds, those with alkyl chains of intermediate size (4C) were the most active. Although the compounds evaluated were less active against the tested tumor cells, they were, in most cases, more selective than the drug doxorubicin. Substances that showed considerable effects on the inhibition of tumoral cell growth and high selectivity could serve as structural models for laboratory synthesis and subsequent in vivo evaluation for potential drug application.

## Figures and Tables

**Table:** Values of growth inhibition (GI_50_) and selectivity index (SI) for alkyl-norstictic acid derivatives and doxorubicin against HT-29 (colon carcinoma), 786-0 (kidney carcinoma), MCF-7 (breast carcinoma), HEP_2_ (laryngeal carcinoma), PC-03 (prostate carcinoma), B16-F10 (murine melanoma), UACC-62 (human melanoma), and NIH/3T3 (mouse embryonic fibroblast) cell lines. SI was calculated as the GI50 value for a compound on 3T3 cells divided by the GI50 value for the compound on a line of cancer cells.

Substances	Cell lines							

	786-0	MCF7	HT-29	PC-03	HEP2	B16-F10	UACC-62	NIH/3T3

**Norstictic acid (1) μM**	758.9^a^	161.7^a^	915.6^a^	191.2^a^	156.9^a^	167.5^b^	88.4^b^	695.9^a^
**SI**	0.9	4.3	0.7	3.6	4.4	>4.1	>7.6	

**8′-** ***O*** **-** ***n*** **-propyl-norstictic acid (2) μM**	140.0	410.0	470.0	30.0	70.0	>600.0	50.0	63.0
**SI**	0.43	0.15	0.13	2.03	0.94	>0.1	1.2	

**8′-** ***O*** **-** ***n*** **-butyl-norstictic acid (3) μM**	28.15	13.26	38.26	6.37	7.94	45.03	11.53	625.20
**SI**	22.21	47.15	16.34	98.11	78.77	13.88	54.1	

**8′-** ***O*** **-** ***n*** **-pentyl-norstictic acid (4) μM**	76.22	15.53	38.20	8.77	48.19	55.40	37.82	57.25
**SI**	0.75	3.69	1.50	6.53	1.19	1.03	1.51	

**8′-** ***O*** **-** ***n*** **-hexyl-norstictic acid (5) μM**	31.4	9.53	7.16	7.23	5.96	56.30	51.6	54.9
**SI**	1.75	5.76	7.66	7.60	9.2	0.97	1.06	

**8′-** ***O*** **-isopropyl-norstictic acid (6) μM**	50.1	59.3	50.17	1.28	7.78	9.65	6.2	43.2
**SI**	0.86	0.73	0.86	33.8	5.56	4.48	7.0	

**8′-** ***O*** **-** ***sec*** **-butyl-norstictic acid (7) μM**	21.38	6.8	12.3	52.40	7.60	9.2	29.4	594.0
**SI**	27.78	87.4	48.3	11.30	78.0	64.4	20.2	

**8′-** ***O*** **-** ***tert-*** **butyl-norstictic acid (8) μM**	63.3	64.2	138.6	7.60	57.8	90.3	67.1	38.5
**SI**	0.6	0.6	0.28	5.0	0.66	0.42	0.57	

**Doxorubicin μM**	0.43	0.086	0.43	0.034	0.65	0.43	0.12	0.55
**SI**	1.23	6.4	1.23	16.0	0.84	1.28	4.5	

a Bogo et al. (2020),

b Brandão et al. (2013).
